# Ichthyol in Skin Diseases

**Published:** 1902-02-15

**Authors:** Arthur Ernest Taylor

**Affiliations:** Assistant Physician Cardiff Infirmary


### HOSPITAL CLINICS AND MEDICAL PROGRESS.

### ICHTHYOL IN SKIN DISEASES.

By ARTHUR ERNEST TAYLOR, M.B., C.M., L.R.C.P., L.R.C.S. Edin., Assistant Physician Cardiff Infirmary.

IN the treatment of various skin affections I can speak most highly of Ichthyol. Cases of eczema in all forms, acne, psoriasis, and numerous other skin affections of an allied character have done well, and in some cases have yielded almost instantaneously to the treatment of Ichthyol internally and externally.

I quote some typical cases :—

(*a*) Hospital patient: Presented himself at my out-patient department suffering from acne vulgaris, chiefly affecting the back, which was of so virulent a character that it was scarcely possible to discern a single patch of healthy skin. Various remedies had been tried with only temporary improvement; at last I decided to try Ichthyol. I gave him it internally and also in the form of an ointment (20 per cent.) to be applied every night. In a few weeks he had greatly improved, and now, after five months' treatment, he is practically well, but I am still continuing treatment as a few spots have shown themselves on his face.

(*b*) This case occurred in my private practice. A female, aged 35, who suffered from pustular eczema of the face which had troubled her for some years. She had been under the care of two London skin specialists but could not, unfortunately, spare the time and expense to carry out the recommendations and treatment advised by them. Knowing that most of the ordinary remedies had been applied, it struck me that I would give Ichthyol a trial. Accordingly, I put her on it—internally and externally. For a few weeks I am bound to confess that my faith in Ichthyol did not increase, but now, after three months' treatment, the result is beyond what I had hoped for. The patient's face is almost clear and she can attend to her duties as head milliner in a large establishment here with comfort, whereas formerly she shrank from them.

I am not prepared to say in either of the two cases I have quoted that the cure is complete, only that results so far convince me that there is great service in the future use of Ichthyol.

(*c*) This patient had been under my care some three years ago for scaly eczema, and in spite of all remedies I knew of improved so slowly that I advised a change of air. He went to Yorkshire and when there placed himself under the care of a well-known physician, and he slowly improved, and was able in three months to return home partly cured, but the complaint never really left him, and was a continuous source of trouble. He is a very keen cricketer, and a very good player, but unfortunately the state of his arm and leg has been a great handicap to him, and kept him out of the field to a great extent. He called early one morning to see me, suffering from strangulated hernia, which I failed to reduce, and operation was decided upon. This was done by my colleague, Dr. Lynn Thomas, and when placing him back in bed, after a most satisfactory operation, my attention was called to the state of his skin. I found that the arms, legs, and trunk were involved in one continuous mass of scaly eczema. With my colleague's consent I promptly gave him Ichthyol internally and instructed the nurses in charge to rub him from head to foot with 20 per cent. Ichthyol ointment. This was done persistently for three weeks whilst he remained in the private hospital, and was afterwards continued by himself. The result was most gratifying, and he stated that he had not been so free from the complaint for years. I examined him a few days ago; the eczema has practically disappeared, and the skin is looking healthy and well.

Some months ago, early in 1901, I was consulted by an elderly gentleman suffering from exfoliative dermatitis supervening on pemphigus. It had troubled him for some time and was undermining his health to a great extent. I put him on many and various remedies, both external and internal, but the case made no improvement. All the ordinary remedies had had a fair trial, but I was getting anxious and the patient more and more irritable at the non-progress. Having had some considerable success with Ichthyol in other cases I determined to try it in this case and see what the result would be.

I may add that the patient was over 65 years of age. I first placed him on gr. v. of Ichthyol internally three times a day and had an ointment of Ichthyol 20 per cent. applied to the feet and scrotum, etc., which were the parts chiefly affected. For the first fortnight the improvement was very slow, but the pain had been reduced. At the end of 14 days I increased the ointment to 30 per cent. and the internal dose to gr. vii. three times a day After another 14 days when the crusts had commenced to dry up and reduce in size, I put him on a still stronger ointment, this time 50 per cent. From this time forward he never looked back, and in six to seven weeks from the starting of the Ichthyol treatment he was able once more to resume outdoor exercise, at first, of course, limited, but early in September he was able to go away to the seaside, where he did a lot of walking and came home at the end of a month perfectly well, able to walk any distance and his weight back to the usual standard. In my own mind I am satisfied that Ichthyol has a great future in the treatment of skin diseases, both acute and chronic.

I have other cases under treatment both in my hospital practice and in private practice, who are being benefited by the use of this drug.

